# Rs6757 in microRNA-3976 binding site of CD147 confers risk of hepatocellular carcinoma in South Chinese population

**DOI:** 10.1186/s12957-022-02724-w

**Published:** 2022-08-17

**Authors:** Fenfen Guo, Hong Li, Lizhong Wang, Xiaoping Song, Jiangfeng Wang, Qingqing Feng, Jinbao Zong

**Affiliations:** 1 Qingdao Hospital of Traditional Chinese Medicine, Qingdao Haici Hospital, No. 4, Renmin Road, Shibei District, Qingdao, 266034 China; 2 The Qingdao Center Hospital, Qingdao, China

**Keywords:** Single nucleotide polymorphism (SNP), CD147, MicroRNA-3976 (miR-3976), Hepatocellular carcinoma (HCC)

## Abstract

**Background:**

Cluster of differentiation 147 (CD147) overexpression plays a key role in the proliferation, differentiation, invasion, metastasis, and prognosis of hepatocellular carcinoma (HCC). The aim of this study was to explore the relationship between rs6757 and the HCC risk in the South Chinese population, and the functional significance of rs6757 by affecting the efficacy of microRNA-3976 (miR-3976) binding to the CD147 3′-UTR.

**Methods:**

We performed a retrospective case-control study to analyze the association between rs6757 and the risk of HCC. We chose candidate microRNAs with the potential of interacting with rs6757 through a series of silico analyses. A luciferase reporter gene assay was implemented to detect the binding extent of microRNAs to each polymorphic allele of rs6757.

**Results:**

An obvious association between rs6757 and the risk of HCC was detected in C vs. T (OR = 1.826, 95% CI [1.263–2.642]), CC vs. TT (OR = 4.513, 95% CI [1.510–13.489]), dominant genetic model (OR = 1.824, 95% CI [1.120–2.965]), and recessive genetic model (OR = 3.765, 95% CI [1.286–11.020]). Bioinformatics analysis indicated that miR-3976 binding sites containing the rs6757-T allele had lower free energies than those with the C allele, the lower free energies, the higher affinities. Luciferase activity was remarkably decreased by miR-3976 binding to the CD147 3′-UTR bearing rs6757 T allele, which could be reversed by miR-3976 inhibitors. Furthermore, miR-3976 reduced the luciferase expression in a manner of dose-dependent when cotransfected with constructs with the CD147-TT-pSICHECK2.

**Conclusions:**

The research we have done suggests that rs6757 confers the CD147 allele-specific translational suppression by miR-3976, which provides a theoretical basis for antineoplastic therapy targeting CD147.

## Introduction

HCC is one of the most common types of all cancers worldwide, and is the fifth most common cancer among men and the ninth most common cancer among women [1, 2]. Globally, HCC is the third leading cause of cancer-related deaths [3]. Although it is commonly recognized that HCC is caused by a combination of genetic, immunologic, and environmental factors, its complete etiology has not been fully understood.

CD147, as well as basigin (BSG), is a transmembrane glycoprotein which regulates the level of matrix metalloproteinase (MMPs) through cell–matrix and cell–cell interaction. Previous work on CD147 have indicated that CD147 is involved in a variety of pathophysiological functions [4]. CD147 is highly expressed in a variety of tumors and metastatic cancer cells [5, 6]. The expression level of CD147 is not only related to the age of the patient and tumor type, but also related to the clinical stage and histopathologic type of the tumor [7]. Disruption of Arf6-mediated CD147 trafficking markedly decreased the migration and invasion of liver cancer cells, while high expression of the Arf6-CD147 signaling components in HCC was closely correlated with poor clinical outcome of patients [8]. Hypophosphorylated CD147 promotes the migration and invasion of HCC cells and correlates with an unfavorable prognosis in HCC patients [9]. TGF-β1 stimulation upregulated CD147 expression and mediated the differentiation of HCC cells. The overexpression of CD147 induced the dedifferentiation and enhanced the malignancy of HCC cells, and also increased the transcriptional expression of TGF-β1 by activating β-catenin [10]. Considering the important role of CD147 in tumors, we speculated that CD147 genetic polymorphisms might influence the occurrence and subsequent development of HCC.

MicroRNAs (miRNAs) are endogenous non-coding RNAs about 22 nt which may regulate gene expression by targeting mRNAs for degradation or translational repression at the post-transcriptional level [11]. The previous work on miRNAs have indicated that miRNAs are associated with many biological processes such as cell proliferation, apoptosis, differentiation, and tumorigenesis [12]. Recently, more and more studies focus on single nucleotide polymorphisms (SNPs) in miRNAs target sites (miRSNPs) which can enhance or weaken miRNA–mRNA interaction s[13–15]. MiRSNPs of susceptibility genes could affect complex traits/diseases through changing the function of miRNAs [16, 17]. Any changes in the recognition site could destroy binding sites or modify binding affinity, which could result in the evasion of miRNA negative regulation [18]. However, until now there have been no studies reported about the association between the miRSNPs of CD147 and the risk of HCC.

Therefore, the purpose of this study was to investigate the association between the rs6757 and the risk of HCC in South Chinese population through a new mechanism of precise gene regulation by miRNA binding ability.

## Materials and methods

### Patients and controls

We performed a case-control study including 155 HCC patients (135 males and 20 females with an average age of 50.7) and 151 healthy controls (98 males and 53 females with an average age of 45.1) on a random basis admitted to the hospital for treatment at the First Affiliated Hospital of Guangxi Medical University from January 2017 to June 2017. All HCC patients were positive for hepatitis B surface antigen (HBsAg) and had never received radiotherapy or chemotherapy. The diagnostic criteria for primary liver cancer are the guidelines for the diagnosis and treatment of primary liver cancer formulated by the Ministry of Health of the People's Republic of China in the 2017 edition. A total of 151 healthy controls were recruited from the Physical Examination Center of the First Affiliated Hospital of Guangxi Medical University during the same period. Inclusion criteria were HBsAg positive; liver function, serum tumor markers, blood lipids, blood glucose, and other test results were normal; and X-ray or B-ultrasound inspection showed no tumors.

### DNA isolation and genotyping

Whole blood samples were collected by vacuum blood collection with EDTA-K_2_ anticoagulant. Genomic DNA was isolated according to the instructions of a Blood Genomic DNA Extraction Kit (Axygen Scientific Inc., USA), and stored at – 80 °C. Primers for the rs6757 were designed using Primer Premier software (version 6.0, Palo Alto, CA). The upstream and downstream primers used were 5′-GCCTGTCTGAAGCCAATGC-3′ and 5′-AACATGCCGAGAGGAGTAACA-3′. The PCR conditions for amplifying targets were predenature 1 cycle of 5 min at 94 °C, then 35 cycles of 30 s at 94 °C, 30 s at 65 °C, and 30 s at 72 °C, followed by 5 min at 72°C. PCR products were directly sequenced to determine the CD147 rs6757 by Genomic Company (Shenggong Biological Engineering Co, Shanghai, China).

### In silico analyses

MirSNP (http://cmbi.bjmu.edu.cn/mirsnp), a database of polymorphisms predicting miRNA–mRNA binding sites, identifies miRNA-related SNPs in GWAS SNPs and eQTLs [19]. Apart from this, we analyzed the miRNA binding sites in CD147 3′-UTR using miRBase (http://www.mirbase.org/index.shtml) and TargetScan (http://www.targetscan.org/). With these bioinformatics tools, we identified hsa-miR-3976 potentially binding to a stretch of sequence harboring the rs6757. Subsequently, we utilized RNAhybrid (https://bibiserv.cebitec.uni-bielefeld.de/rnahybird) to predict the miRNA-target binding structures and energies.

### Plasmid construction and luciferase assay

The TT genotype of rs6757 was amplified by PCR using CD147-TT-F: 5′-ccgctcgagGGCAGGTGGCCCGAGGACGC-3′ and CD147-TT-R: 5′-ataagaatgcggccgcGAGGGTGGAGGTGGGGGCGA-3′, which were tagged with the *XhoI* and *NotI* restriction enzyme sites (underlined) respectively. The rs6757 TT genotype inserts were digested by *XhoI/NotI*, gel-purified, extracted, and then subcloned into the *XhoI/NotI* site of pSICHECK2 vector (Promega). The prepared plasmid was designated as CD147-TT-pSICHECK2. The inserts were sequenced to verify the validity and orientation relative to the luciferase gene at Genomic Company (Shenggong Biological Engineering Co, Shanghai, China). Using the site-directed mutagenesis, we constructed CD147-CC-pSICHEC-K2 that contains rs6757 CC genotype sequence by using CD147-CC-F: 5′-GAGGACGGCCGGCTCTCCATAGCACCAGGGCTCACGTGG-3′, and CD147-CC-R: 5′-CCACGTGAGCCCTGGTGCTATGGAGAGCCGGCCGTCCTC-3′ primers. The inserts of CD147-CC-pSICHECK2 were also confirmed by sequencing.

### Cell culture

HEK293T cells (from Shenggong Biological Engineering Co.) were cultured with high-sugar DMEM medium containing 10% fetal bovine serum at 37 °C in humidified 5% CO_2_.

### Transfection and luciferase assay

2 **×** 10^4^ cells per well were seeded in 96-multiwell plates. After the cells reached 50–60% confluence, we used Lipofectamine 2000 reagent (Invitrogen) to transfect the prepared CD147-TT-pSICHECK2 or CD147-CC-pSICHECK2 (500 ng), 50 nmol/L hsa-miR-3976 mimics, hsa-miR-3976 control (as a control for hsa-miR-3976, the specific sequence is: UCACAACCUCCUAGAAAGAGUAGA), 100 nmol/L hsa-miR-3976 inhibitor (reverse sequence of hsa-miR-3976), and 100 nmol/L hsa-miR-3976 negative control (as a control for hsa-miR-3976 inhibitor, the specific sequence is UUUGUACUACACACAAAAGUACUG) into the cells. Over and above these, various concentrations (1.0, 2.5, 5.0, and 10 nM) of hsa-miR-3976 mimics were added to the transfection system to observe the effect of hsa-miR-3976 on luciferase activity at different concentrations. Each transfection was carried out in triplicate. The data were analyzed by normalizing firefly luciferase activity with that of the Renilla luciferase.

### Statistical analysis

Continuous variables were compared using an unpaired t-test. The differences of alleles and genotypes frequency distribution between the groups were statistically analyzed using the *χ*^2^ test. The Hardy–Weinberg equilibrium (HWE) was tested by a *Q* test with one degree of freedom [14, 15]. The association between CD147 rs6757 and the risk of HCC was estimated using the odds ratio (OR) and the 95% confidence interval (CI). After adjustment for age, gender, smoking, and drinking, adjusted ORs and 95% CIs were calculated by logistic regression. The value of relative luciferase activity were presented as means ± SD and analyzed by Student’s *t* test. All statistics were performed by SPSS software version 19.0 and Stata software version 11.0. It was considered to be statistically significant when *P* < 0.05.

## Results

A total of 155 HCC patients and 151 controls were enrolled in this study. There were significant differences in gender, age, and drinking status between HCC patients and controls. However, no significant difference was found regarding smoking (Table 1).

**Table 1:** Baseline characteristics of the HCC and control group

Variables	HCC	Control	*P value*
Age, years	50.7 ± 11.0	45.1 ± 11.6	0.000
Gender, *n* (%)			
Male	135 (87.1)	98 (64.9)	0.000
Female	20 (12.9)	53 (35.1)	
Smoking, *n* (%)			
Yes	75 (48.4)	62 (41.1)	0.197
No	80 (51.6)	89 (58.9)	
Drinking, *n* (%)			
Yes	54 (34.8)	36 (23.8)	0.035
No	101 (65.2)	115 (76.2)	

### Association of the CD147 rs6757:T>C with HCC susceptibility

The T/C variant (rs6757) was genotyped by sequencing in all 306 participants (Table 2). As shown in Table 2, the frequencies of the rs6757 genotypes and alleles between the HCC patients and controls were statistically significant (*χ*^2^ = 10.721, *p* = 0.005; *χ*^2^ = 10.364, *p* = 0.001). The distributions of genotypes in controls and HCC patients were compatible with HWE (*P*_Controls_ = 0.558, *P*_HCC_ = 0.351). The detailed results are shown in Table 3. After adjusting for confounding factors including age, gender, smoking, and drinking, significant associations between the T/C variant (rs6757) and the risk of HCC were detected in C vs. T (OR = 1.826, 95% CI [1.263–2.642]), CC vs. TT (OR = 4.513, 95% CI [1.510–13.489]), dominant genetic model (OR = 1.824, 95% CI [1.122–2.965]), and recessive genetic model (OR = 3.765, 95% CI [1.286–11.020]). C variant (rs6757) was considered to be an independent risk factor for HCC. The detailed results are shown in Table 4.

**Table 2:** The distribution of CD147 rs6757 in HCC and control group

		HCC (*n* = 155)	Control (*n* = 151)	*χ*^2^	*P* value
Genotype, *n* (%)	CC	18(11.7)	5 (3.6)	10.721	0.005
	CT	62(39.7)	51 (33.8)		
	TT	75(48.6)	95 (62.6)		
Allele, *n* (%)	C	98(31.6)	61(20.2)	10.364	0.001
	T	212(68.4)	241(79.8)

**Table 3:** The HWE of CD147 rs6757 genotypes

Groups	Genotype			*HWE*
	CC	TT	CT	
HCC	18	75	62	0.351
Control	5	95	51	0.558

*Abbreviations*: *HWE* Hardy-Weinberg equilibrium

**Table 4:** The association between the rs6757 and risk of HCC

		HCC (*n* = 155)	Control (*n* = 151)	OR(95CI)	*P* value
Genotype, *n* (%)	TT	75(48.6)	95(62.6)	1	
	CT	62(39.7)	51(33.8)	1.562(0.939–2.598)	0.086
	CC	18(11.7)	5(3.6)	4.513(1.510–13.489)	0.007
Allele, *n* (%)	T	212(68.4)	241(79.8)	1	
	C	98(31.6)	61(20.2)	1.826(1.263–2.642)	0.001
Dominant genetic model, *n* (%)	TT	75(48.6)	95(62.6)	1	
	CT + CC	80(51.4)	56(37.4)	1.824(1.122–2.965)	0.015
Recessive genetic model, *n* (%)	CT + TT	137(88.3)	146(96.4)	1	
	CC	18(11.7)	5(3.6)	3.765(1.286–11.020)	0.016

Adjust confounding factors including age, gender, smoking, and drinking

*Abbreviations*: *CI* confidence interval, *OR* odds ratio

### Rs6757 is located in the predicted binding sites of hsa-miR-3976 in the CD147 3′-UTR

**Fig. 1:**
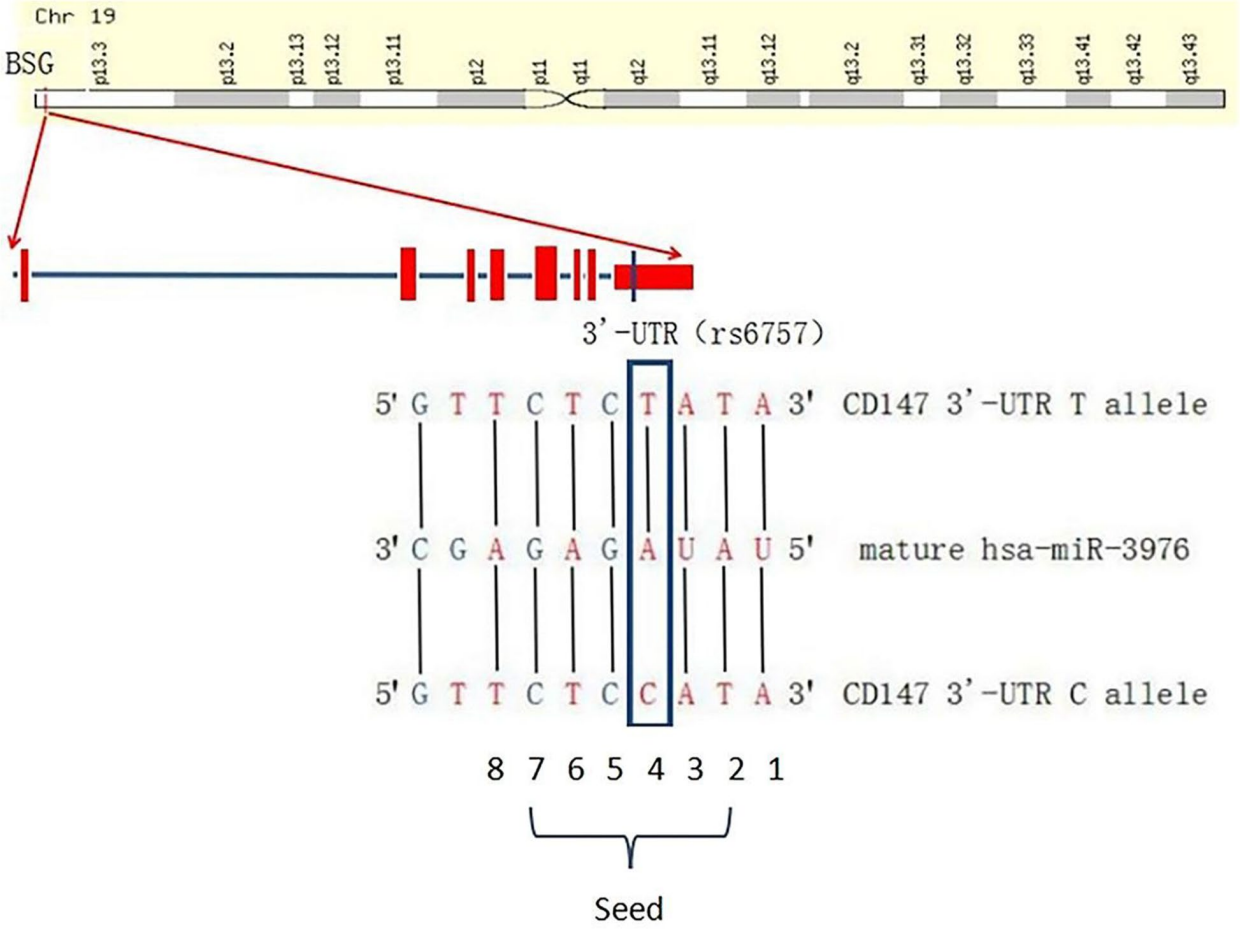
Combination of different alleles of rs6757 to hsa-miR-3976. The T/C variant (rs6757) is located in the seed region of miRNA-3976, which means that the C/T variant (rs6757) has the potential to disrupt the binding of miR-3976 to CD147 3′-UTR

With the development of bioinformatics, there are more and more websites or software tools for predicting miRNA target genes online. Through integrating and analyzing the prediction results of different websites or software such as MirSNP (http://cmbi.bjmu.edu.cn/mirsnp), miRBase (http://www.mirbase.org/index.shtml), and TargetScan, we found that there were multiple potential miRNAs in the CD147 3′-UTR region. After intersecting these predicted results and comparing them in the NCBI database, we found that the rs6757 was exactly located in the seed region of has-miR-3976 (Fig. 1).

**Fig. 2:**
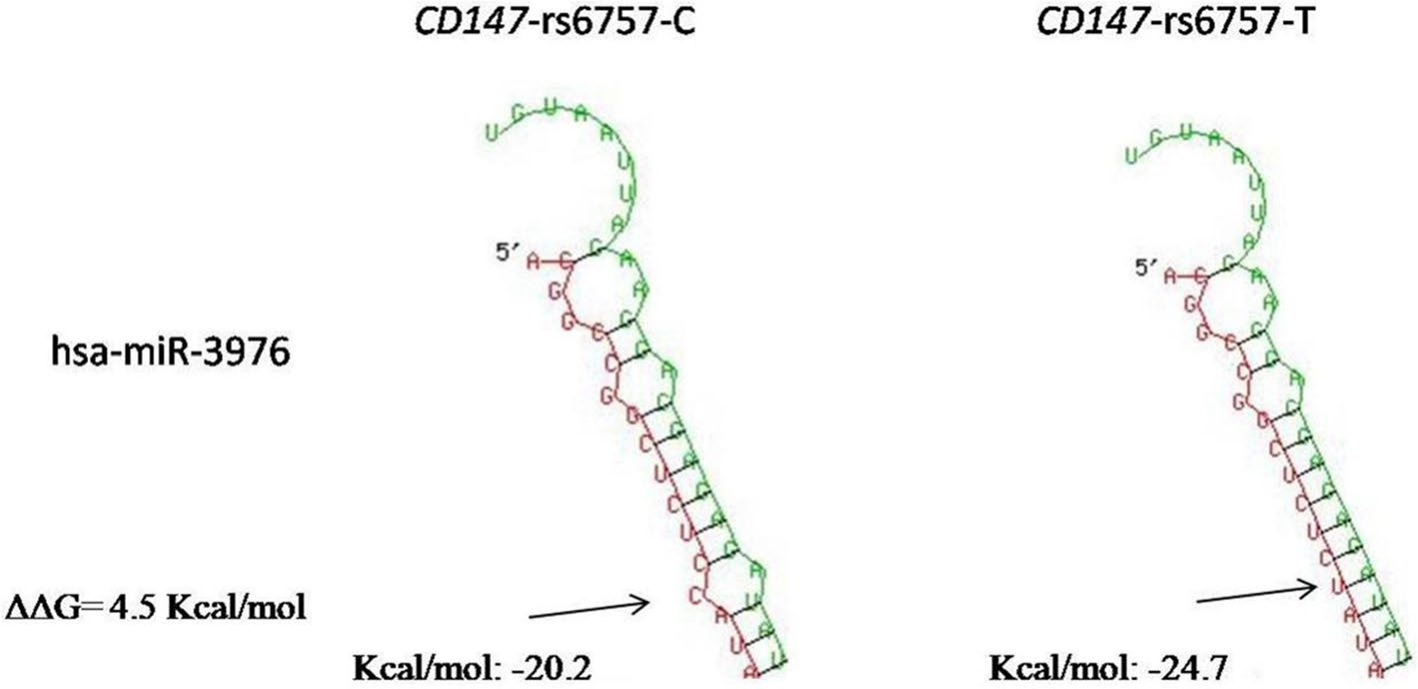
In silico analysis of the pairing of miR-3976 to the binding site in the 3′-UTR of CD147. The arrow indicates the SNP site in the CD147 3′-UTR in each folding structure. The lower minimal folding energies, the more stable binding; the higher the sum of the ΔΔG value, the more likely to be a functional SNP

The forecasted minimal folding energies of the hsa-miR-3976 target duplexes for the rs6757-T and -C alleles were − 24.7 kcal/mol and − 20.2 kcal/mol respectively (Fig. 2). Obviously, the minimal folding energies of the duplexes rs6757-T-containing (− 24.7 kcal/mol) is lower than that of the duplexes rs6757-C-containing (− 20.2 kcal/mol). The lower minimal folding energies, the more stable binding of hsa-miR-3976 to the CD147 3′-UTR, which indicated more efficient translational repression for the CD147 transcript containing rs6757 T variant. The predicted sum of the ΔΔG value was 4.5 kcal/mol, which suggested that the rs6757 was more likely to be a functional SNP.

### Functional analyses of rs6757

**Fig. 3:**
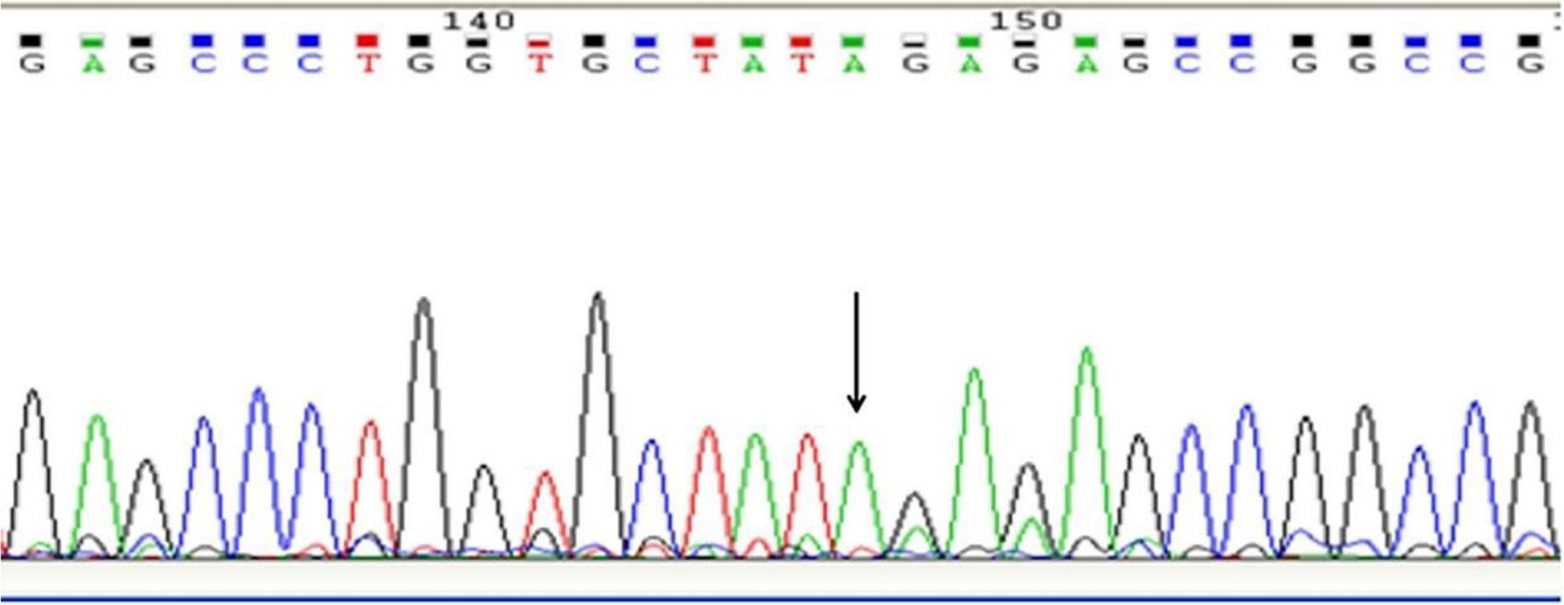
Sequencing results of the inserts in CD147-TT-pSICHECK2 plasmid. Arrows indicate the inserted rs6757 TT genotype fragment

**Fig. 4:**
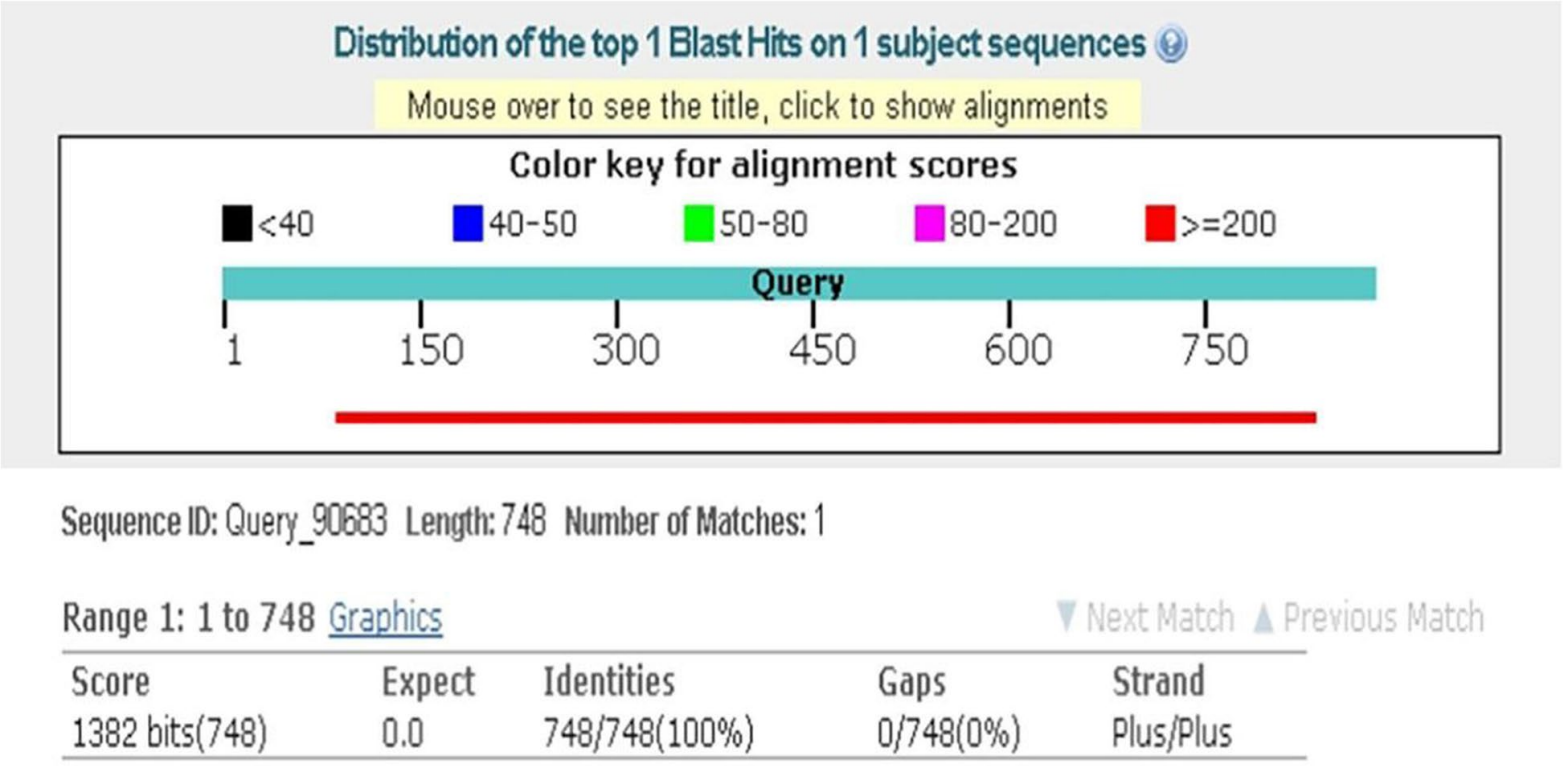
The Blast of the inserts in CD147-TT-pSICHECK2 plasmid. The sequencing results were analyzed by Blast, the inserted sequence of target gene was 100% consistent with the sequence of CD147 rs6757 TT genotype in the NCBI database

**Fig. 5:**
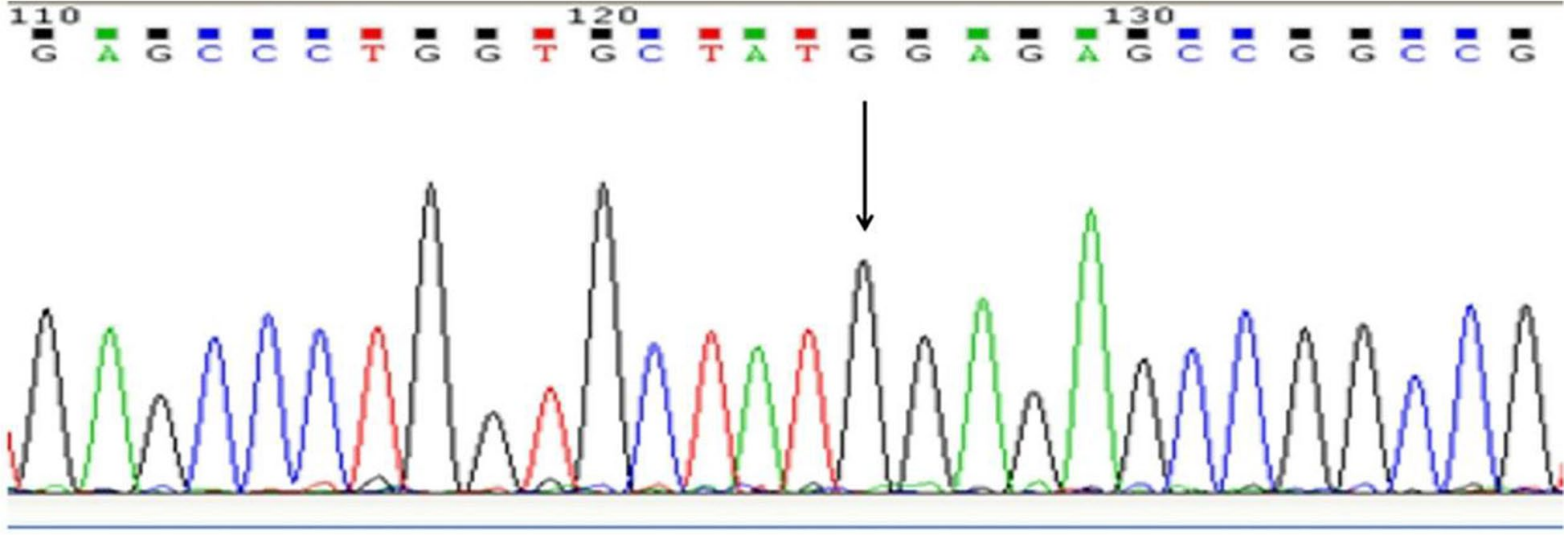
Sequencing results of the inserts in CD147-CC-pSICHECK2 plasmid. The sequencing result showed that the mutation from nucleotide T to C at the target site (marked in green) of CD147 gene had successfully achieved

**Fig. 6:**
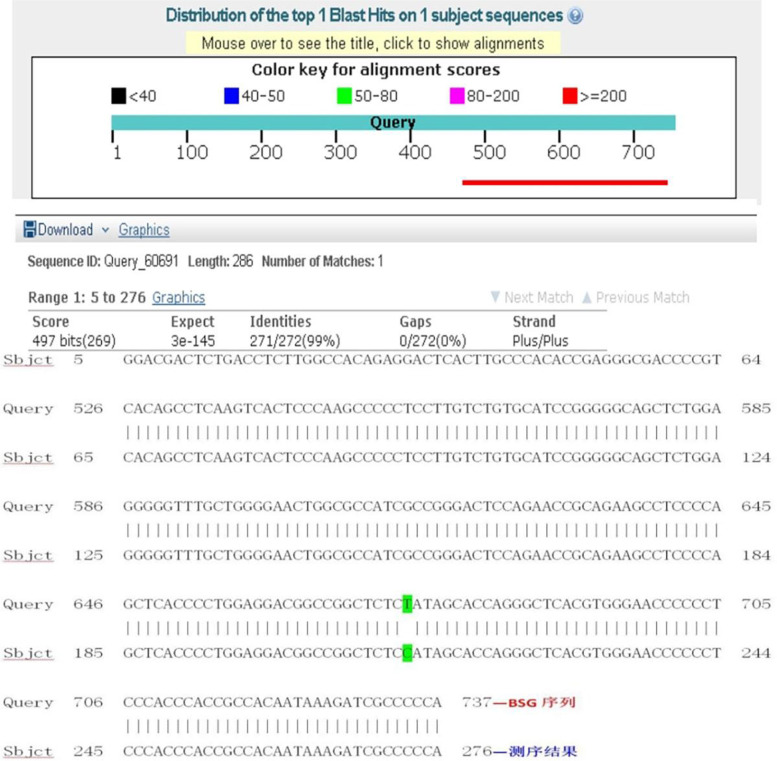
The Blast of the inserts in CD147-CC-pSICHECK2 plasmid. After mutating from nucleotide T to C, the inserted sequence was 99% consistent with the sequence of CD147 in the NCBI database by Blast analysis

**Fig. 7:**
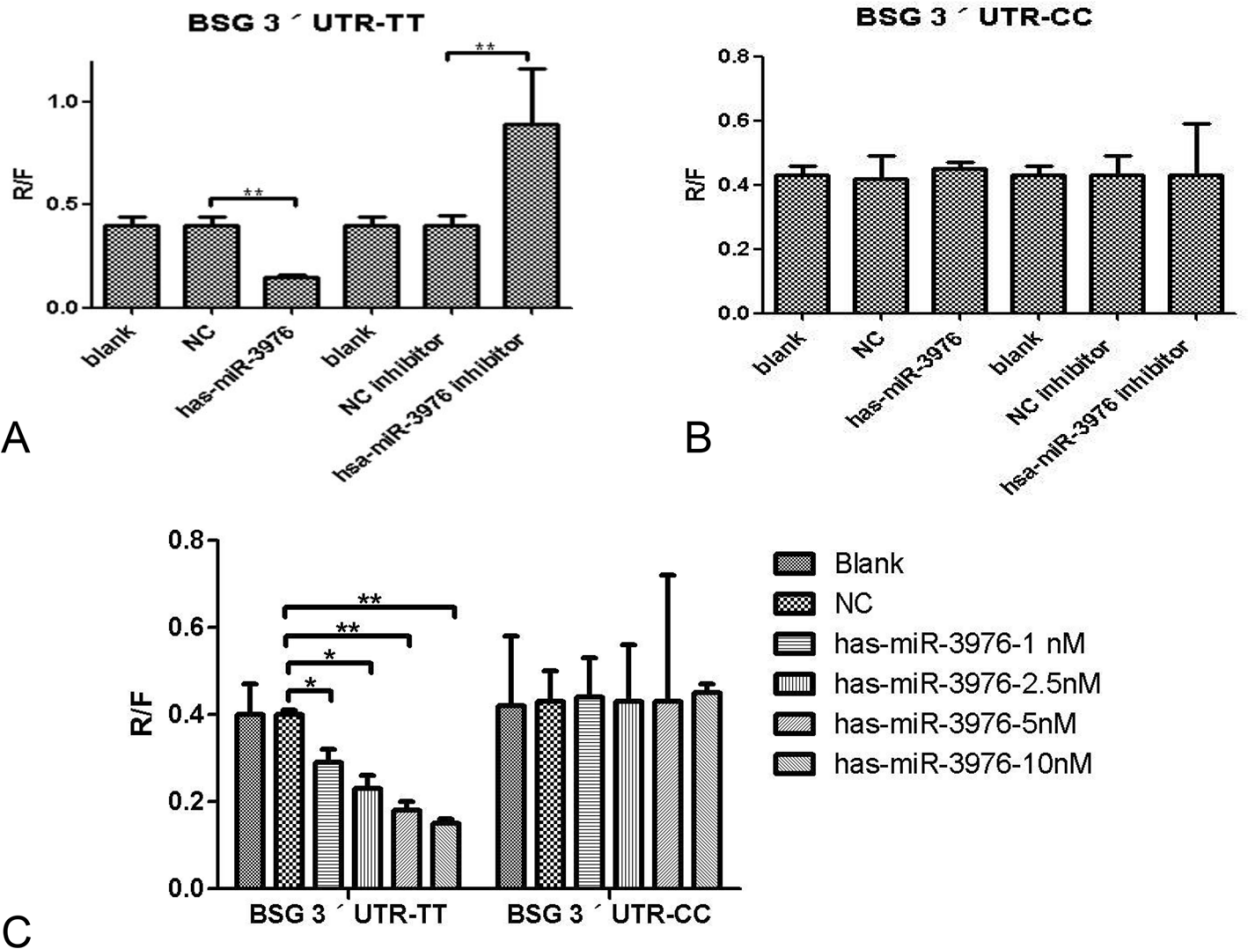
The R/F analysis of a luciferase reporter vector. **A** Luciferase expression was significantly reduced following transfection with CD147-TT-pSICHECK2 (*P <* 0.01); the translational suppression of miR-3976 can be reversed by its inhibitor. **B** There was no significant statistical difference between the groups following transfection with CD147-CC-pSICHECK2. **C** Luciferase activity was decreased by miR-3976 in dose-dependent manner for the constructs with a TT genotype but not changed for constructs with a CC genotype at the rs6757:T>C polymorphism. Each transfection was carried out in triplicate. * denotes a *p* value *<* 0.05; ** denotes a *p* value *<* 0.01

In order to detect whether the T/C variant (rs6757) affected the CD147 translational regulation by affecting the binding of miR-3976 to CD147 3′-UTR, we constructed the luciferase reporter vectors containing rs6757 different genotypes (Figs. 3, 4, 5, and 6). Luciferase expression was significantly reduced following transfection with CD147-TT-pSICHECK2 (*P <* 0.05); this translational suppression of miR-3976 can be reversed by its inhibitor (Fig. 7A). However, there was no significant statistical difference between the groups following transfection with CD147-CC-pSICHECK2 (Fig. 7B). Not only so, miR-3976 dose-dependently reduced the luciferase expression when cotransfected with constructs with the CD147-TT-pSICHECK2 (Fig. 7C). However, miR-3976 showed no effect on the luciferase expression when cotransfected with constructs with the CD147-CC-pSICHECK2 (Fig. 7C).

## Discussion

In accordance with the principle of genetic diversity, differences in evolution and environment result in differences in genes and phenotypes between different populations [20, 21]. Therefore, it is important to study the relationship between these genetic variants and genetic susceptibility of different people. To our knowledge, no study has been conducted on rs6757:T>C and HCC risk in the South Chinese population.

In this research, we calculated the associations between the rs6757 and risk of HCC in 155 HCC patients and 151 controls. We find an obvious relationship between C/T variant (rs6757) and the genetic susceptibility of HCC, participants carrying allele C had a distinctly increased risk of HCC (OR = 1.826, 95% CI [1.263–2.642]; *P* = 0.001).

As shown in Fig. 1, the T/C variant (rs6757) is located in the seed region, nucleotides 2−7, of miRNA-3976, which means that the C/T variant (rs6757) has the potential to disrupt the binding of miR-3976 to CD147 3′-UTR. We predicted and calculated the minimal free energies of the secondary structures of miR-3976 bounding to the CD147 3′-UTR containing T/C variant (rs6757) by computational modeling. The minimal folding energy and the ΔΔG value were used to assess the impact of SNPs on the binding ability of the miRNA with its target mRNA [22]. The minimal folding energies of the duplexes rs6757-T-containing (− 24.7 kcal/mol) is lower than that of the duplexes rs6757-C-containing (− 20.2 kcal/mol). The lower minimal folding energies, the more stable binding of miR-3976 to the CD147 mRNA. The sum of the ΔΔG value was 4.5 kcal/mol, the higher sum of the ΔΔG value of the rs6757, the more likely to be a functional SNP.

As expected, luciferase reporter gene assay showed that miRNA-3976 suppressed the luciferase construct bearing the TT, but not the CC, genotype of the rs6757 (Fig. 7). Our investigation confirm that the C/T variant (rs6757) has an obvious effect on miR-3976 binding affinity, which causes greater suppression when miR-3976 bounding to CD147 3′-UTR containing the T allele rather than the C allele.

Despite many new discoveries mentioned above, there are some limitations in this study. First, although we selected health individuals without signs or symptoms of HCC as the control group, it should be noticed that the controls did not undergo a CT or MRI plain scan. Second, the sample size in the present study was still not large enough to reveal genes with allele frequencies between 5 and 10% [23]. Third, taking into account geographic variations, the prevalence of C/T variant (rs6757) in the South Chinese population may bias the results based on the single-center case-control studies. Fourth, which confounding factors should be selected for multivariate analysis has always been a controversial problem. Generally, most researchers choose the following two methods to solve this problem. One is that only dissimilar confounding factors should be corrected. The other suggests all confounding factors should be corrected. In this paper, we adopted the second approach. Actually, no matter which method you choose, the statistical results have no significant change. Finally, We simply verified that the rs6757:T>C would affect on the binding of miR-3976 to CD147 3′-UTR using the luciferase reporter assay in HEK293T cells. Next, we will study the relationship between the rs6757 and HCC in vitro at least two liver cell lines.

To sum up, we have revealed that CD147 is a direct target of miR-3976, and the C/T variant (rs6757) could affect on the binding of miR-3976 to CD147 3′-UTR, which result in a statistically significant association between the rs6757 and risk of HCC in the South Chinese population. However, further studies are needed to explore the new mechanism of subtle gene regulation of CD147 through miR-3976 binding capacity affected by rs6757.

## Data Availability

Not applicable.
